# A Smart Sensor for Defending against Clock Glitching Attacks on the I2C Protocol in Robotic Applications

**DOI:** 10.3390/s17040677

**Published:** 2017-03-25

**Authors:** Raúl Jiménez-Naharro, Fernando Gómez-Bravo, Jonathan Medina-García, Manuel Sánchez-Raya, Juan Antonio Gómez-Galán

**Affiliations:** Department of Electronic Engineering, Computers, and Automation, University of Huelva, Ctra Huelva-La Rábida, s/n, 21819 Huelva, Spain; naharro@diesia.uhu.es (R.J.-N.); jonathan.medina@diesia.uhu.es (J.M.-G.); msraya@diesia.uhu.es (M.S.-R.); jgalan@diesia.uhu.es (J.A.G.-G.)

**Keywords:** smart sensor for robots, hardware vulnerability, mobile robot attack, clock signal defense

## Abstract

This paper presents a study about hardware attacking and clock signal vulnerability. It considers a particular type of attack on the clock signal in the I2C protocol, and proposes the design of a new sensor for detecting and defending against this type of perturbation. The analysis of the attack and the defense is validated by means of a configurable experimental platform that emulates a differential drive robot. A set of experimental results confirm the interest of the studied vulnerabilities and the efficiency of the proposed sensor in defending against this type of situation.

## 1. Introduction

Over the last recent years, the study of vulnerability in electronic devices has attracted the attention of the scientific community [1,2,3,4]. Among electronics systems, robotic platforms are one of the most widely used today. Currently, robots are responsible for executing many critical tasks such as rescue, surveillance, industrial processes, and different operations in general day life [5,6,7,8].

Robot control is traditionally implemented by a computer-based architecture, so the study of security issues regarding computer vulnerability represents a relevant matter to develop secure robots. Security analysis involves the study of the effects of voluntary actions with the aim of violating or modifying the system behaviour. These types of actions are usually known as attacks, and the weakness exploited by the attacker is known as a vulnerability.

Moreover, not only it is important to detect vulnerabilities, but also to define mechanisms for a feasible defense. Some strategies have been developed for defending computed systems. On the one hand, masking the transmitted information in order to preserve communication privacy can be a defense objective; several methodologies have been applied for this purpose, like for instance the use of encryption algorithms [9] or synchronized chaotic systems [10]. On the other hand, another strategy is to develop a device to detect the attack and avoid the intentions of the attackers [1,2].

This paper deals with the study of a specific vulnerability that affects mobile robots and presents the design of a sensor for defending against this particular type of attack. Many different works have addressed the study of vulnerability of computer-based systems from the point of view of software [11,12] and hardware [1,2,3,13]. The attacks that exploit the hardware vulnerabilities are known as hardware attacks.

Although reliability and fault tolerance of robots are open issues recently discussed by the scientific community [14,15], the vulnerability of robotic systems has not been frequently considered. Hardware and software attacks directly decrease robots’ reliability.

The study of the effect of software attacks can be easily extended to a robotic platform. However, the effects of hardware attacks should be particularly considered, by taking into account the specific sensors and actuators applied in the robotics field [16,17].

An important question regarding this matter is the communication between the control system and the peripherals. In this context, the Inter-Integrated Circuit (I2C) protocol is a commonly used mechanism to establish this communication process. In fact, the use of I2C low level controllers in mobile robotics has spread in the last years. I2C is a synchronous protocol, where the clock signal plays a relevant role. Clock synchronization heavily depends on the applied synchronization protocol and algorithm; a detailed overview of the state of the art about this matter can be found in [18,19,20].

This paper aims to discuss the possibility of attacking a robotic system by interfering with the clock signal of the I2C protocol and proposes a defense strategy for this attack. Though this work is focused on the I2C protocol, other different types of protocols can also be objectives of clock attacking. In fact, the strategy presented in this work is based on detecting anomalies in the clock frequency. Therefore, this solution is suitable for defending any protocol vulnerable to an attack that injects frequency variations in the clock signal, as for instance: CAN-bus, SPI protocol, etc.

The case study presented in this work is related with autonomous mobile robot navigation. Namely, it is supposed that the robot navigates by applying a path tracking algorithm, so that it is capable of visiting certain areas of interest defined by the user. Typical mobile robotics applications use this type of navigation strategy [5,6,21,22]. In this context the paper studies the possibility of attacking the communication process between the high level controller and low level controller using the I2C protocol, and evaluates the efficiency of the proposed defending device.

The paper is organized as follows: Section 2 is devoted to presenting the basis of clock attacking and to explain vulnerabilities of the I2C protocol. Section 3 shows details about the proposed countermeasure strategy. Section 4 introduces the case study and describes the experimental platform. In Section 5 several experimental results that validate the authors’ hypothesis are shown. Finally, some conclusions are drawn in Section 6.

## 2. The Clock Signal: A Possible Source of Attacks

The main objective of many attacks is the interference with the clock signal due to its importance in the system operation. More particularly, the perturbation may consist in modifying the frequency or the period of the clock signal.

**Figure 1:**
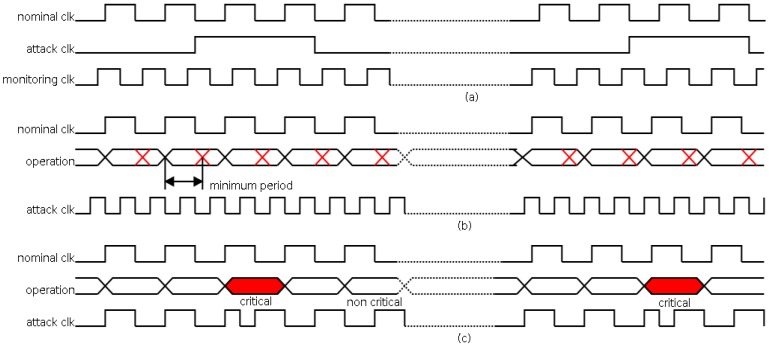
Examples of attack using clock signal. (**a**) Attack increasing the clock period; (**b**) attack decreasing the clock period; (**c**) attack to a certain instruction: clock glitching attack.

In Figure 1, several attacks using clock signals are shown. In this figure, signal *nominal clk* is the clock signal free of attack (with the correct period), and signal *attack clk* is the clock signal with attack (with the altered period).

In Figure 1a, an attack increasing the clock period is shown. The outcome of this attack is a decrease in the operation speed. This interference can be used, for instance, to monitor the main signals (using the *monitoring clock* signal) for applying reverse engineering [23]. In Figure 1b, an attack decreasing the clock period is shown. The decrement must be lower than the minimum period to guarantee the correct operation (as seen from its minimum period). The effect of this attack is a global malfunction of the system that can be used, as an example, to cause a denial of service. In Figure 1c, an attack decreasing the clock period, focused on a certain operation, is shown. In this case, the effect of the attack consists in avoiding a certain operation (as seen from the delay of this operation). This attack is usually known as a clock glitching attack.

This last attack can be used, for instance, against cryptocircuits implementing an encryption algorithm. The aim of the attack is to avoid the complete cycle of the encryption causing a vulnerability in the circuit. This fact can be achieved independently on the implementation platform (processor, ASIC, FPGA, …) because all of them require a clock signal. This situation is discussed in [24].

### 2.1. Attack on the Protocol I2C

I2C vulnerability has not been frequently addressed [25,26]. This protocol is widely used in robotics platforms to connect the controllers with actuating elements like motors and sensors. This section is devoted to the study of clock glitching attacks in the I2C protocol. They are easy to implement, and very difficult to detect (this fact increases the danger), so it is important to study the effect and the solutions to this threat in a so frequently used protocol.

This protocol uses two different signals: a *scl* signal that has the synchronization information; and a *sda* signal that has the data information. The communication process involves the transmission of three different types of data: slave address, with a maximum of 128 slaves (or 1024 slaves in the extended version); register address, indicating the register to interact; and register value, indicating the transmitted value. One of the main characteristics of this protocol is the signaling: the high logic level is in a situation of high impedance; and the low logic level is the reference voltage. Therefore, the communication involves the connection of ground to *scl* and *sda* signals in the low logic level case. Due to the high impedance, both master and slave can write in *scl* and *sda* signals. Also, the high impedance is a source of attack because any element can access and write on *sda* and *scl* signals without causing any conflict.

**Figure 2:**
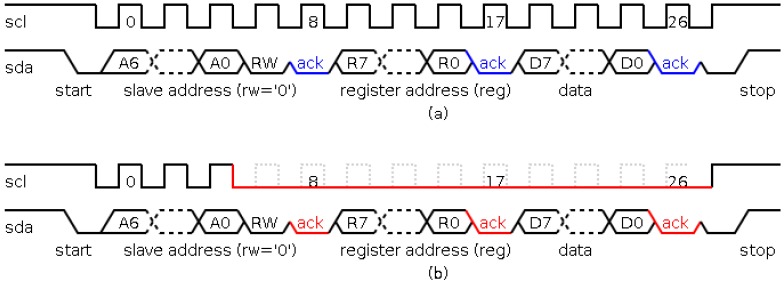
Behavior of I2C protocol. (**a**) Write operation; (**b**) attacked write operation.

As an example of the behavior of the I2C protocol, Figure 2a shows a write operation (the black color identifies that the value is written by a master, and blue color identifies that the value is written by a slave). This operation works as follows. Firstly, the communication begins in idle state (identified by *scl* and *sda* signals to the high level). The communication is controlled by the master and starts with a start condition (identified by a falling transition in the *sda* signal while the *scl* signal is high). Next, the address of the slave to communicate is sent (*A6:A0* signals). After that, *RW* signal is sent (the write operation is identified by a low level signal). Following, the slave sends an acknowledgement (identified by a low level in the *sda* signal in the 8th *scl* pulse) indicating that the slave has acknowledged its participation in the communication process. The next step is to send the register address of the slave in which the master will write the transmitted value (*R7:R0* signals), and the slave sends the acknowledgement in the 17th pulse of the *scl* signal. Finally, the master sends the value that will be written in the register of the slave, and the slave acknowledges the writing operation (in the 26th pulse of the *scl* signal). The communication process finishes with a stop condition (identified by a rising transition in the *sda* signal while the *scl* signal is at a high level recovering the idle state).

The characteristics of this protocol allow to perform focused attacks, i.e., to attack the communication with a particular slave or the communication with a certain register of a specific slave. In this paper, the attacks involve the variation of the period in the *scl* signal of the protocol. An example is shown in Figure 2b. This figure illustrates an attack in the communication process of a certain slave (the black color identifies the value written by the master, and the red color identifies the value written by the attacker). In this process, three modules are involved: a master, a slave and the attack module.

The communication process begins normally, that is, in the start state with the transmission of the slave address. Till this moment, the behavior of the attack module consists in monitoring the communication in order to decide when to perform the attack, depending on the transmitted address. If the attack must begin, the clock signal is flattened, i.e., it is forced to keep a low value during the communication process. Then, the attack module writes a low level in the *scl* signal, and as a consequence, no *sda* signal value will be seen by any slave module, thus, the message will be lost. Before that, the attack module generates an acknowledgement signal so that the master does not identify an anomalous situation.

## 3. Countermeasures

If a system responsible of a critical task is at risk of being attacked, a defense strategy must be included in the system design. The main purpose of this strategy should consist in thwarting the objectives of the attacker. When a vulnerability source is the clock signal, any defense strategy should include a frequency sensor in order to detect anomalous period values. In this approach, clock glitching attacks are detected by means of this type of sensor. A particular implementation of a digital frequency sensor is cited in [27]. As mentioned in that paper, the standard detection solutions are usually based on analog filters, such as the one included in [28]. The comparison between that solution and the use of a digital frequency sensor has been also presented in [27]. That result is not affected by the modifications implemented on the sensor in this new application.

**Figure 3:**
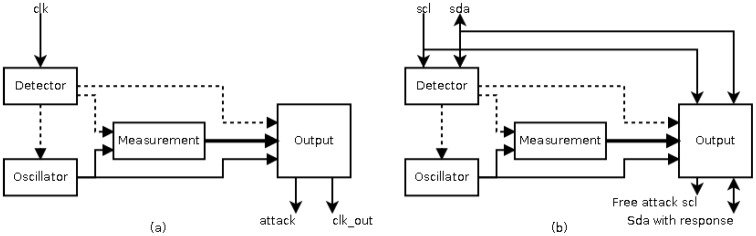
Architecture of the frequency sensor as countermeasure against an attack based on clock signal. (**a**) Basic architecture in [27]; (**b**) New architecture adapted to I2C protocol.

Although the sensor can be materialized by using VLSI [27] or FPGA techniques, in this paper a FPGA implementation has been considered so that a rapid prototype is achieved. The basic architecture of this sensor is shown in Figure 3a. The sensor consists of four blocks: a transition detector, to detect a new transition during signal monitoring and to begin the measurement of the frequency; a local oscillator, to avoid similar attacks on the sensor; a measurement block, to measure the frequency as pulses of the local oscillator; and an output block, to generate the response of the sensor. The response of this sensor is to regenerate the clock signal when its frequency is out of the allowed range. Though the concept of the sensor presented in [27] is valid for the situation shown in Figure 2 (the attack to the I2C protocol), its implementation is not valid for different reasons:

- The idle state of the protocol (with a frequency equal to zero) would be identified as an attack.
- The low frequency of some slaves requires excessive hardware resources in the implementation of the local oscillator.
- The response of the sensor is the same than the objective of attack, that is, to flatten the *scl* signal. Therefore, a new response must be generated.

Then a new implementation for the detector of transition, the local oscillator, and the output blocks must be done.

### 3.1. Transition Detector

**Figure 4:**
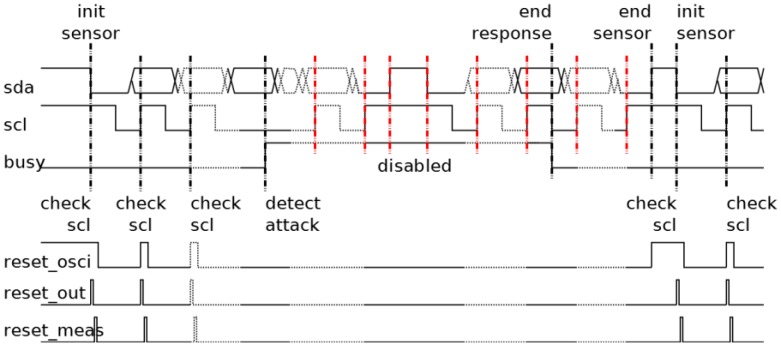
Behavior of the transition detector. The following cases are considered: a start condition; a detected attack; a stop condition; and a new start condition.

The main function of the transition detector is to identify the beginning of a new operation, a transition of the *scl* signal. However, while in the early implementation, the reset signals are only active due to the transition (see Figure 4); the actual implementation maintains a reset condition during the idle state in the transmission, and so, the idle state is not considered as an attack.

The behavior of the transition detector is shown in Figure 4. During the idle state of the protocol, the sensor waits for a new communication process resetting the local oscillator. This waiting finishes when a start condition arrives. In this moment, the reset cycle is completed and a new measurement begins. Following, all *scl* signal pulses are measured by the sensor until the arrival of a stop condition or the detection of an attack. In the case of a detected attack, the sensor would activate its response (*busy* signal is activated). During the response, the transition detector is disabled until the response has finished (*busy* signal is deactivated) and the current communication process finishes (the arrival of a stop condition). Following that, the local oscillator is reset until a new start condition arrives.

**Figure 5:**
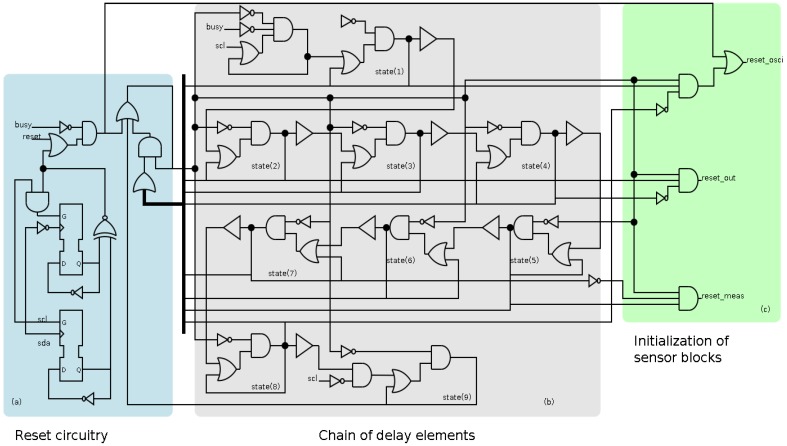
Schematic of the transition detector, identifying the three main sections: (**a**) reset circuitry; (**b**) the chain of delay elements; and (**c**) the initialization of the sensor blocks (local oscillator, measurement block and output block).

The early behavior is implemented asynchronously because the clock signal is flattened during its operation. The implementation of the transition detector consists of three sections, shown in Figure 5. Firstly, there is a reset circuitry whose main function is the initialization of the block. This section identifies the arrival of a new event in the *scl* signal and the start and stop conditions. Secondly, a chain of delay elements whose main function consists in generating the adequate delays. Thirdly, a section to generate the initialization of the rest of sensor blocks with the adequate sequence.

It is worth mentioning that the chain of delay must guarantee that the sequence of the reset signals of the different blocks is correct. This sequence is detailed in [27], and a short description is as follows: first the oscillator is reset, then the output block is reset and activated, after that the measurement block is reset and activated, and finally, the oscillator is activated. In this case, the sequence requires ten delay elements. The main component of the delay element is a buffer (shown in Figure 5), which is implemented by using a transparent latch.

### 3.2. Local Oscillator

Though the function of this block is independent on the application, the use of low frequency can require an excessive number of resources (basically flip-flops). A comparison in hardware resources considering an upper limit of frequency of 12.5 kHz is shown in Table 1. This table shows a comparative study concerning the number of flip-flops used in different implementations of the sensor. The differences are due to the different implementations of the local oscillator generating different periods.

The total number of flip-flops is determined by the sum of the number of flip-flops in the following building blocks: the local oscillator, the transition detector, the measurement block and the output block. In the case of the local oscillator, this number is equal to the relation between its period and the delay of one element. In the case of the transition detector, the number is fixed and equal to 8. In the case of the measurement block, the number is equal to the relation between the upper limit of the allowed period and the oscillator period. In the case of the output block, the number is equal to the half of the flip-flops of the measurement block plus the necessary elements to implement the I2C order (that is fixed).

Table 1 shows that the implementation with a minimum number of flip-flops involves a trade-off between a low and a high frequency in the local oscillator. As a consequence of these reasoning, two alternatives can be used: a high frequency local oscillator, with a high number of flip-flops in the measurement and output blocks; or a low frequency local oscillator, with a high number of flip-flops in the local oscillator. However, the optimal solution involves a trade-off between a low and a high frequency in the local oscillator.

**Figure 6:**
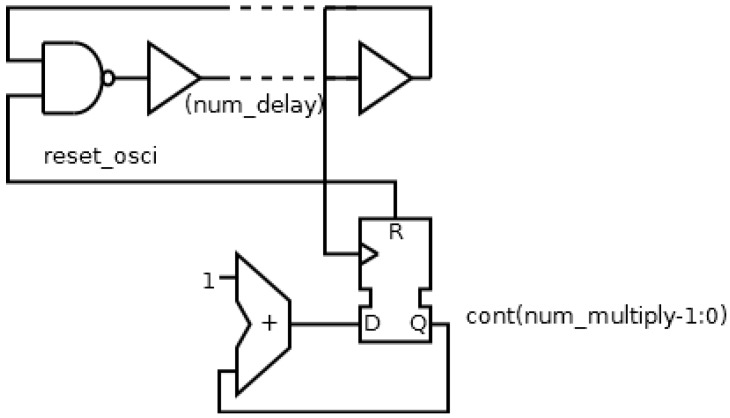
New implementation of the local oscillator based on a ring oscillator and a frequency divider.

Though it is possible to find an optimal configuration, the minimum number of flip-flops is considerable. A new implementation is going to be designed in order to reduce the hardware resources. This new solution consists of two different strategies: a ring oscillator and a binary counter as frequency divider; as shown in Figure 6. The ring oscillator will generate a precision period, while the frequency divider will multiply the period without necessity of many hardware resources. Therefore, the new implementation will use two configuration parameters: the number of delay element in the ring oscillator (*num_delay*), and the number of the flip-flops in the divider (*num_multiply*).

The following step will be to compare the two implementations. This comparison is shown in Table 2, where *delay_element_* is the delay of one delay element, *num_delay* is the number of delay elements and *num_multiply* is the number of counter bits. Therefore, the period of the local oscillator and the number of flip-flops in the local oscillator are shown in (1):

period of local oscillator = [(*delay_element_* + *delay_routing_*) * *num_delay*] * 2^*num_multiply*^, number of flip-flops = *num_delay* + *num_multiply*, (1)

The first equation indicates that the period of the local oscillator depends on the period of the ring oscillator (expression between the brackets) multiplied by the frequency divider factor (expression at the right of the brackets). Also, the number of flip flops depends on the number of elements needed for the implementation of both the ring oscillator and the frequency divider.

Table 2 shows a study of the optimal configuration of the oscillator (using a ring configuration determined by *num_delay* and a frequency divider determined by *num_multiply*). Three configurations for a same nominal period are considered: minimizing *num_delay*; minimizing *num_multiply*; minimizing the sum of *num_delay* and *num_multipy*. Due to the quantization of the period with the delay elements, the period obtained cannot be equal to the nominal period, and hence there will be an error between the nominal and the obtained period. The optimal configuration will be a trade-off between hardware resources (sum of *num_delay* and *num_multiply*) and error, that is, the third configuration: minimizing the sum of parameters.

### 3.3. Output Block

The main function of the output block is to generate the response of the sensor, which has to be particularly defined in each application. In this case, the response is going to be divided into two different actions. Firstly, when no attack is detected, the response of the sensor will be to allow the passage of the protocol signals (*sda* and *scl* signals). On the other hand, when an attack is detected, the response of the sensor will be to disconnect the slave from the *sda* and *scl* attacked channel and to induce a certain defense behavior in the slave in order to avoid the possible objectives of the attacker.

**Figure 7:**
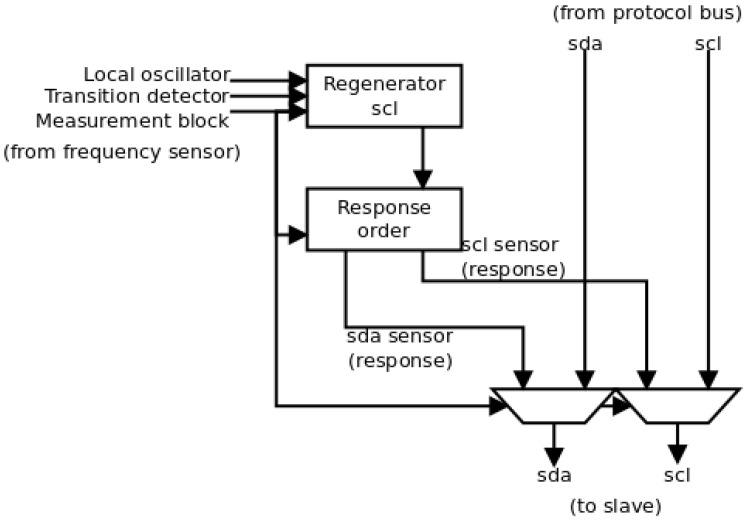
New implementation of the output block based on the multiplexing of the bus signals.

A block scheme of the output block is shown in Figure 7. There, the two different actions can be identified. The selection between both actions comes from the measurement block that identifies the arrival of an attack. Firstly, the pass of the protocol signals is identified by the direct connection to the multiplex units. This direct pass avoids possible desynchronizations between both the *scl* and *sda* signals.

In the second case, depending on the location of the sensor, the attack response can be different. On the one hand, if the sensor is on the same substrate (and hence in the same security region), the sensor will have direct access to the register included in the I2C transmission. Therefore, it will be able to write directly a certain value in the register in order to induce the defense behavior. On the other hand, if the sensor is in the same security region but on a different substrate, it does not have direct access to the register. Therefore, the response of the sensor will be sending a secure clock signal and a specific sequence of I2C messages in a particular order, so that the defense behavior is initiated. In this case, the sensor will supplant the master functions. In this paper, the second option is applied.

In Figure 7, this strategy is implemented in two different blocks. Firstly, the sensor must regenerate the *scl* signal from the master to maintain the same timing as in the rest of transactions. This regeneration is done in the *scl regenerator* element. This element is similar to the element in the sensor referenced in [27], and it needs information from the local oscillator (the period of the local clock signal), the transition detector (to initiate its operation) and the measurement block (the period of the original *scl* signal and the signal to start the sensor order). Once the *scl* signal is regenerated, the *sda* signal is generated to send the defense order by the *response order* element. This order can represent one or several I2C transactions (because it may be necessary to write in several registers of the slave). The transactions must include the address of the slave, the address of the registers and the value to write in the registers. The behavior of this element is fixed, and hence, the only information that it needs comes from the measurement block (the signal to initiate the transaction).

### 3.4. Sensor Simulations

The elements described above have been implemented in a FPGA device using a VHDL model. The use of a ring oscillator involves that the clock period depends on the delay of the ring, and hence, it is necessary a post-routed simulation to consider the delay of the implementation. The implementation requires the use of a determined device, and in this work, a Spartan 3AN700 device is considered. However, the VHDL model allows implementing this design in any platform (any FPGA device or a VLSI system).

**Figure 8:**
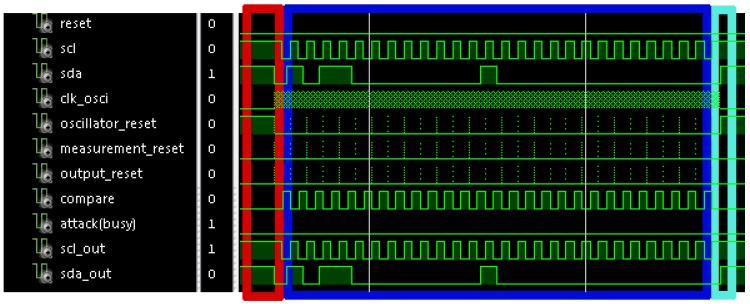
Waveform of a simulation of a normal I2C communication process with no attack. This communication is performed between the master and the slave whose address is X”B0”. The master will write the value X”10” in the register X”00”.

Figure 8 shows a simulation of a normal transaction with no attack. In this figure, the oscillator reset is active during the idle state and the sensor begins the initialization sequence when a start condition arrives. This sequence begins again when a new cycle of *scl* signal begins. No attack is identified because the *attack* (*busy*) signal is not activated. In this case, *sda_out* and *scl_out* signals are copies of the *sda* and *scl* signals to avoid synchronization problems in the communication.

**Figure 9:**
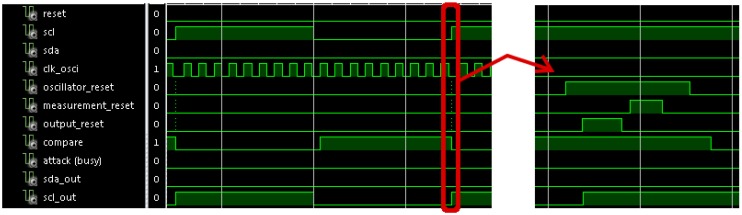
Waveform of a zoom of the simulation shown in Figure 8, and a detail of the initialization sequence.

Figure 9 shows a zoom of the waveform shown in Figure 8. Concretely, it shows the operation of a cycle in *scl* signal. It can be appreciated that the *compare* signal is active when the period enters the allowed range, and it is deactivated when a new cycle arrives such that it is ready for a new verification. Also, it shows a detail of the initialization sequence in *resets* signals.

**Figure 10:**
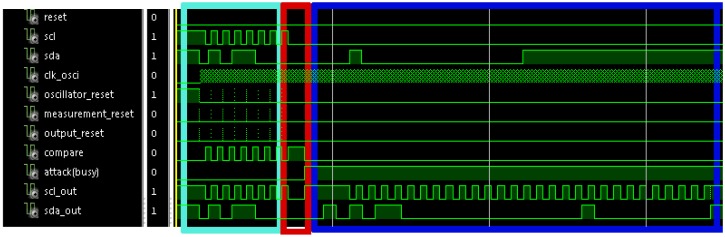
Waveform of a simulation of an attacked I2C communication process with the implemented defense.

Figure 10 shows the simulation of a communication process with an attack in cycle number eight. In it, the first seven cycles have the correct period, and hence, *scl* and *sda* signals pass to *scl_out* and *sda_out* signals. The 8th cycle does not arrive because the attacker wants to disable the communication. This action is identified because *attack* signal is activated, and hence, the sensor sends the response to the slave. This response begins by sending a stop condition, so that the slave closes the previous communication process, and starts listening the *sda* signal in order to check if it is the receiver of the following communication. After that, a start condition and the address X”B0” (corresponding to the secured slave) are sent. Following, the register address is sent (X”00”); and finally the value to write is sent (X”10”). Once data have finished, the sensor sends a stop condition to finish the communication process.

## 4. Case Study: the Navigation of a Mobile Robot

This section illustrates a real case of attack against the clock of an I2C communication process in a robotic system. Later sections will clearly demonstrate the efficiency of the proposed sensor in defending real systems against this type of attacks. The interest in attacking robots is justified by the use of these systems in a huge variety of contexts. The application presented in this paper is related with mobile robot navigation along a previously defined path. It represents a typical situation in many of the current robotic applications (industry, agriculture, service, etc.), performed whether in outdoor or indoor scenarios [21,22,29,30].

It is supposed that user’s intentions involve visiting certain areas of interest by the robot. For this purpose, a planning algorithm has provided a path that goes through these areas. It is also assumed that a path-tracking algorithm is applied so that the robot follows this trajectory with precision.

Hence, those who want to interfere with the user’s intentions would try to modify the course of the robot, but ensuring that the user does not realize the robot is under attack (apart from the course modification). Accordingly, the planned path should remain invariable, and the external interference may be applied to the low level actuator, for a short period of time, so that the disturbance takes place temporarily leaving no traces.

The following sections describe the details of a real implementation of the aforementioned situations. Attacking and defending strategies have been tested in a platform that emulates a mobile robot allowing the authors to validate their hypothesis about clock hacking.

### 4.1. A robotic Experimental Platform

**Figure 11:**
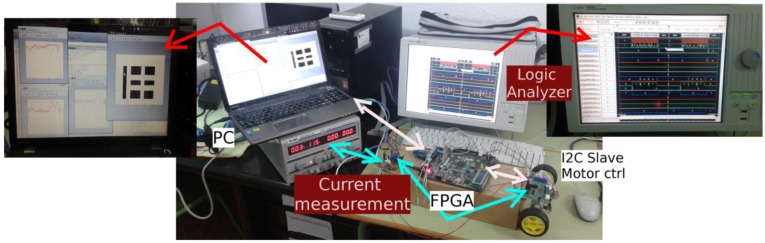
The experimental platform.

An experimental platform has been built in order to emulate the motion of a differential drive robot in virtual environments, so that digital and analogical instrumentation can be used in order to measure and characterize the behaviors of the hardware against attacks (see Figure 11).

In [31] authors have detailed the characteristic of the platform. It implements a control architecture that includes a high level controller and a low level controller. The high level controller is the responsible of taking decision about the robot motion, as well as to set the correspondent actions to be executed by the actuators. In the case of mobile robot applications, the high level controller defines the motors’ speed. Then, these values are transferred to the low level controller so that motors make the robot move in a correct way. In the current application, the high level controller is based on a very well-known path tracking algorithm: Pure Pursuit [21,22,29,32], and the low level control implements a traditional PID controller that transmits orders to the motors driver by using I2C protocol.

**Figure 12:**
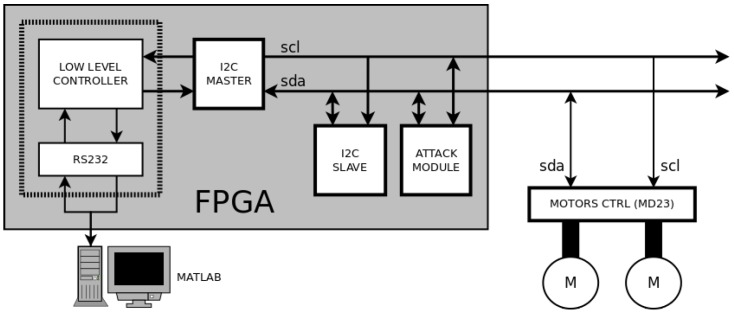
Scheme of the experimental platform.

The experimental platform is divided into three different zones (see Figure 12): a Personal Computer (PC) that executes Matlab software routines to implement the high level controller; a FPGA device to implement hardware modules (such as the low level controller and the attack module), and a standard slave device (more concretely an MD23 motor controller [33]).

The FPGA device is aimed to implement the hardware modules to obtain a better control of the signals. These modules are an UART based on the RS232 protocol (to communicate with Matlab in a PC), a low level controller (to adapt the orders from Matlab to the rest of modules), an I2C master (to control the communication through I2C protocol), an I2C slave (to test the system inside the FPGA device) and the attack module (to implement the attacks to I2C communications). The platform also includes several measurement elements, highlighted in red in Figure 11: a logic analyzer that monitors the main signals in the communication process, the same PC that monitors the velocity of the motors, and a current measuring device (between the supply source and the motor controller) that monitors the current consumed by the motors.

The Matlab application has been configured to work with different virtual scenarios and predefined paths. The application receives information from the FPGA regarding the velocity of the motors, and simulates the motion of the robot within the virtual scenario. The high level controller executes an iterative loop that considers this virtual motion and defines control references so that the robot follows a specific path accurately.

Communications with the FPGA are performed by the RS232 protocol. The UART implemented in the FPGA receives the speeds references from the PC, and encodes and inserts them in the I2C bus through the I2C master. This module generates SCL and SDA signals as information goes out the FPGA to the motors driver. This driver reads the orders by I2C protocol and changes the speed of the motors consequently. The MD23 controller allows measuring the angular velocities of the motors axes. This value is sent from motors’ driver to the I2C master. The low level controller encodes these values, which are sent to the PC. In Matlab, this information is translated to Cartesian motion by applying dead reckoning techniques, so that the position of the robot is estimated. Hence, new reference values for the motors speeds are generated by the path-tracking algorithm that takes into account the new estimated position and the reference path. This flow is repeated until the robot arrives to the final position of the path. Obviously, this platform is a perfect scenario to test the attacking and defending techniques described in Section 2 and Section 3.

### 4.2. Attacking and Defending Strategies

#### 4.2.1. Attacking Strategy

The main idea is to attack the clock signal of the I2C bus in order to interfere with the task of the robot. In this application, the path that the robot follows has been generated in order to ensure that the vehicle goes over some places in a certain order. As a consequence, a first objective for an attacking strategy would be to modify the course of the robot. At a selected moment, when the master tries to communicate with the engines, the attack module acts on the clock, preventing such communication. After that, it sends the acknowledgment. Due of this fact, the attack achieves two effects:

- The master module believes that there has been no problem and that the motor follows the references.
- The slave module receives nothing and maintains the previous speed value.

The consequences of these effects can be dramatic. While the path tracking algorithm orders the robot to follow a collision-free trajectory, the vehicle could go straight ahead and crash into an obstacle. Another way to carry out the attack consists of modifying the robot trajectory without endangering the robot (avoiding possible collisions) but preventing it from correctly performing its task. This most elaborate situation is the one authors have considered. According to this criteria, a successful attack must meet the following set of constraints:

(1) Both during and after the attack, the robot should keep navigating in a safe way.
(2) The consequence of the attack is one of these options:
  (a) The robot will not reach a specified location.
  (b) The robot will repeat the route to visit a certain area.

All of these requirements can be accomplished by an appropriate attack that follows the prescriptions detailed in Section 2.1. As it will be shown in the experimental section, a properly executed attack has a high likelihood of succeeding. The success only depends on selecting the optimum moment for implementing the clock attack. Reverse engineering techniques can be used to determine the moment and duration of the clock signal interference.

#### 4.2.2. Defending Strategy

**Figure 13:**
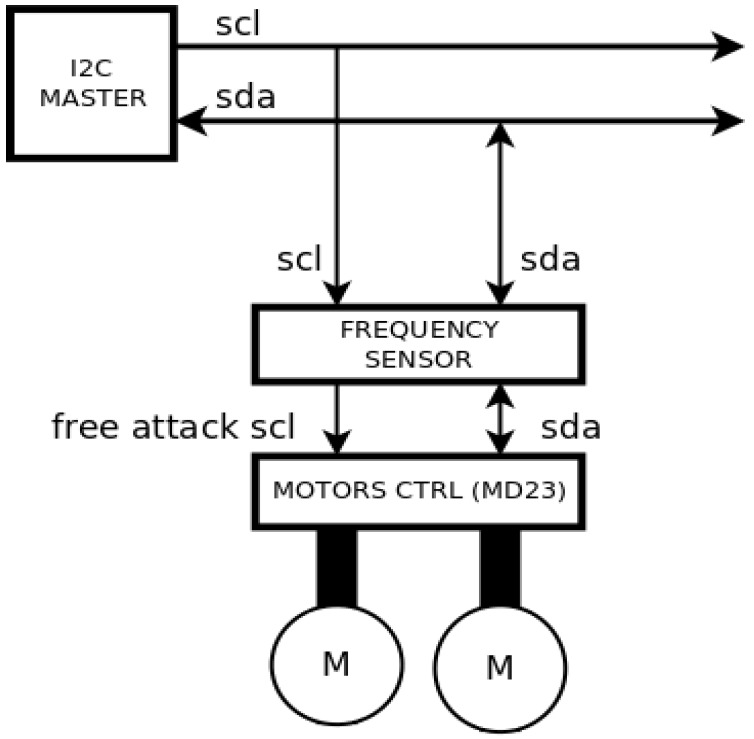
Scheme of the defending strategy.

The strategy for avoiding this type of attack consists on developing a motor driver that incorporates a sensor similar to the one presented in Section 3 (see Figure 13). Each time the sensor detects a clock attack, it is programmed to generate a sequence of speed commands that order the motors to stop. Once the clock attack has finished, the sensor detects that the clock signal is free of interferences and makes the slave follow again the commands received from the I2C master. Then, the robot can start its navigation again and safely accomplish the predefined tasks.

## 5. Experimental Results

Many experiments have been performed in different virtual environments. The idea was to find those types of paths and scenarios in which temporary attacks can accomplish the constraints defined in Section 4.2. In [31], it was demonstrated that an appropriate selection for the attack moment results in a modification of the robot trajectory that prevents it from accomplishing a predefined task.

**Figure 14:**
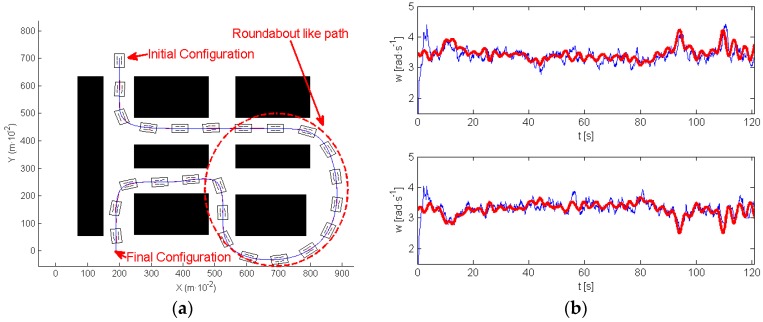
Experiment without attack: (**a**) planned and followed trajectory; (**b**) wheels velocity references and signals from the encoders.

One of the most interesting results was found in the scenario of the experiment shown in Figure 14a. This figure indicates with arrows the initial and final positions. In this experiment, the robot evolves describing what can be called a roundabout-like trajectory, in which the robot moves around a specific area (marked also in Figure 14a). In this first experiment, navigation was implemented without any attack, the path-tracking algorithm succeeded in controlling the motion of the robot very close to the planned trajectory. Figure 14b illustrates, in a red line, the reference for the wheels’ speed, provided by the high level controller, and in a blue line, the wheels’ speed provided by the encoder signals (up right motor, down left motor). 

**Figure 15:**
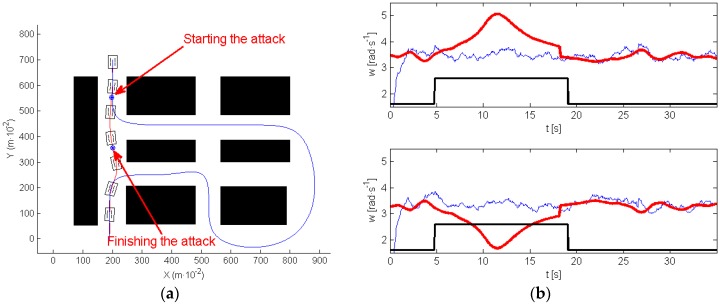
Avoiding the roundabout: (**a**) planned and robot trajectory; (**b**) signal from the encoders.

In the following experiment (see Figure 15), an attack was implemented on both motors. Figure 15a shows in red the path executed by the robot. Figure 15b represents the wheels’ velocity references (red), and the wheels’ velocity provided by the encoder signals of both motors (blue). A black line delimits the moment of the attack. In this case, the clock attack took place before navigating around the roundabout, and disappeared at the moment when the robot was closer to the end of the roundabout. During the attack, the robot navigates straight ahead, and exits the roundabout.

Note that, during the attack (see Figure 15b) the high level controller sent references to the motors in order to make robot move to the closer section of the path, however, and due to the attack, none of the motors listened to these commands. When the attack finished the robot is closer to the final section of the path. The tracking algorithm makes the robot move to this section, and then, it follows the path correctly to the end. As a consequence, the attack was successful because it prevents the system from visiting the roundabout that was one of the tasks defined by the initially planned path.

**Figure 16:**
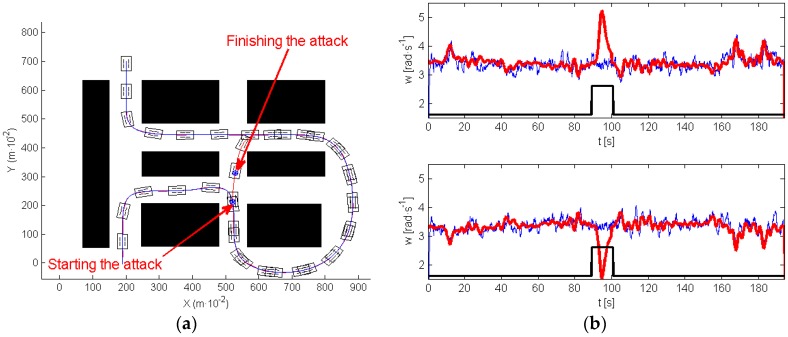
Visiting the roundabout twice: (**a**) planned and robot trajectory; (**b**) signal from the encoders.

In the next experiment, authors have extended the application of this strategy so that the robot keeps navigating around the roundabout instead of avoiding it (see Figure 16). Figure 16a shows in red the path executed by the robot, and Figure 16b represents the velocity references (red) and the wheels’ velocity provided by the encoder signals of both motors (blue). The attack is indicated by the black line.

The clock attack took place when the roundabout was finishing. From this moment, the robot navigates straight ahead. The attack finished when the robot was close to the beginning of the roundabout, then the path tracking algorithm makes the robot follow the roundabout again, visiting this area twice.

These results confirm that a convenient selection for the moment of the attack can make robot change its expected behavior. Nevertheless, the resulting motions appear to be “natural”, in fact the robot navigates safely and returns to the desired trajectory. It is only evident by the bizarre behavior of the motors that probably no one would understand without knowing of the existence of the attack.

**Figure 17:**
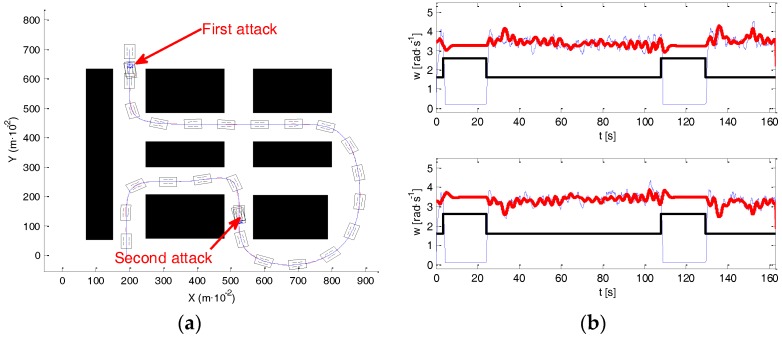
Avoiding the roundabout: (**a**) planned and robot trajectory; (**b**) signal from the encoders.

The next experiment illustrates the efficiency of the proposed sensor in defending against the previous situations. As was explained in Section 4.2, in this experiment the frequency sensor was connected between the MD23 and the I2C bus, protecting the motors’ drivers from the attacks. The robot suffered two attacks at the same moments they were performed in the experiments of Figure 15 and Figure 16. Figure 17a presents the trajectory of the robot in the attacked/defended experiment, showing that the vehicle accurately follows the desired path. Figure 17b shows the velocity references (red), with the wheels’ velocity provided by the encoder signals of both motors (blue) and the attack evolution (black). Observe that, when an attack appears, the velocity references generated by the path tracking algorithm are different from zero. However, the velocity of the motors evolves to zero—remember that motors under attack obey only the commands generated by the sensor and the high level controller does not perceive that any attack has been performed. Once, the attack disappears, motors listen correctly the references of the high level controller and start moving again, accurately following the path.

## 6. Conclusions

This paper pays attention to illustrate an example of attacks on the clock signal in the I2C protocol, and proposes a defense strategy. Particularly, the effect of a clock glitching attack over the communication process between master and slaves has been analyzed. The authors propose the design of a new sensor that represents an effective defense against this type of perturbation. Attacks and defense strategies have been validated in an experimental platform that emulates a differential drive mobile robot. Several experiments concerning mobile robot navigation have been performed. Some particular circumstances have been characterized where I2C clock glitching attacks dramatically increase the robot vulnerability. Experiments show the efficiency of the proposed sensor in detecting and defending the robot from clock attacks.

## Figures and Tables

**Table 1:** Study of the flip-flop numbers varying the period oscillator considering a delay element of 1.91 ns. The upper limit of period is 80 μs (12.5 kHz).

Oscillator Period	Flip-Flops				Total FF
	Oscillator	Detector	Measurement	Output	
4 ns	3	8	20,000	10,000	30,003
40 ns	21	8	2000	1000	3021
90 ns	48	8	889	445	1390
200 ns	105	8	400	200	713
400 ns	210	8	200	100	518
900 ns	472	8	89	45	614
4 μs	2095	8	20	10	2133

**Table 2:** Study of the flip-flop numbers varying the period oscillator considering a *delay_element_* of 1.91 ns. The upper limit of period is 80 µs (12.5 kHz).

Nom. Period (ns)	Ring-Divisor Oscillator			Error Period (%)	Ring Oscillator		Error Period (%)
	*num_delay*	*num_multiply*	Period (ns)		*num_delay*	Period (ns)	
4	1	1	3.82	4.5%	2	3.82	4.5%
400	105	1	401.1	0.3%	210	401.1	0.3%
	1	8	489.0	22%			
	3	6	366.7	8.3%			
4000	1048	1	4003	0.07%	2095	4001.5	0.04%
	1	12	7823	96%			
	4	9	3912	2.2%			
